# MicroRNA-1 suppresses proliferation, migration and invasion by targeting Notch2 in esophageal squamous cell carcinoma

**DOI:** 10.1038/s41598-018-23421-3

**Published:** 2018-03-26

**Authors:** Wenzhi Liu, Mengkao Li, Xiangming Chen, Shan Zhu, Hailong Shi, Dawei Zhang, Cheng Cheng, Baosheng Li

**Affiliations:** 1 Department of Clinical Oncology, Taian City Central Hospital, Taian, Shandong Province P. R. China; 2 Department of Radiation Oncology, Shandong Cancer Hospital Affiliated to Shandong University, Jinan, Shandong Province P. R. China; 3 Shandong Academy of Medical Sciences, Jinan, Shandong Province P. R. China; 4 Department of Neurosurgery, Taian City Central Hospital, Taian, Shandong Province P. R. China; 5 Department of Oncology, Shandong Provincial Western Hospital, Jinan, Shandong Province P. R. China; 6 Trauma orthopedics ward, Zibo Central Hospital, Zibo, Shandong Province P. R. China; 7 Cardiovascular department ward, Zibo Central Hospital, Zibo, Shandong Province P. R. China

## Abstract

MicroRNAs play an important role in the migration and invasion of tumors, and lower expression of microRNA-1 (miR-1) has been proven in a variety of malignant tumors, including esophageal squamous cell carcinoma (ESCC). In this study, we found that miR-1 expression levels in tumor tissues and preoperative serum from esophageal carcinoma patients were lower than those in non-tumorous tissues and healthy volunteers. miR-1 expression in tissues and plasma was closely related to invasion, lymph node metastasis and TNM staging. Additionally, miR-1 expression levels in tissues and plasma were positively correlated. miR-1 inhibited cell proliferation, migration and invasion. Overexpression of miR-1 in ESCC cells reduced Notch2 protein but not mRNA levels, whereas suppression of miR-1 led to an increase in Notch2 protein but not mRNA levels. A dual-luciferase experiment validated that Notch2 was a direct target of miR-1. Introducing Notch2 mRNA into cells over-expressing miR-1 partially abrogated the effects of miR-1 on migration and invasion. Further studies verified that miR-1 regulates EMT signalling pathways directly through Notch2. Therefore, these results confirm that, as a tumor suppressor gene, miR-1 may be a potential tumor marker for the early diagnosis of ESCC and a new drug target.

## Introduction

Esophageal cancer is the world’s eighth most malignant tumor and has a 5-year-survival rate of less than 15%^1^. ESCC, which is characterized by invasiveness, recurrence and metastasis, is the most common pathological type of esophageal cancer in East Asia. Due to the lack of typical clinical symptoms and effective techniques for early diagnosis, esophageal cancer is typically at a late stage when diagnosed. Therefore, it is very important to understand the mechanisms of the occurrence and development of esophageal cancer at the molecular level and to explore diagnostic options and effective treatment targets for early diagnosis and treatment of esophageal cancer.

MicroRNAs (miRNAs) are a class of non-coding RNAs that are approximately 19–22 nucleotides in length and play multiple roles by binding to the 3′-untranslated region (3′-UTR) of target genes^2,3^. More than 60% of human protein-coding genes are expected to be regulated by miRNAs^4^, which are involved in the development and progression of malignant tumors by acting on different target genes^5,6^. These genes play important roles in regulating cell differentiation, proliferation, invasion, apoptosis and angiogenesis^7–9^. Our microarray analysis showed that the expression levels of miR-1 in ESCC tissues were 0.18 times higher than in normal tissue and that miR-1 is a tumor suppressor. In the previous study, miR-1 is downregulated in ESCC^10^, which occurs through the repression of MET, cyclin D1 and CDK4 expression, and indicates a novel strategy for the diagnosis and treatment of ESCC^11^. However, the key target gene of miR-1 in ESCC is still undefined and requires further exploration.

The Notch signalling pathway is a highly conserved pathway that affects cell proliferation, differentiation, apoptosis, and adhesion and is closely related to embryonic development, angiogenesis and tumor formation. The Notch signalling pathway is composed of Notch receptors, DSL protein ligands and intracellular effector molecules. Mammalian Notch receptors can be divided into four types: Notch1, Notch2, Notch3 and Notch4. Notch2 plays an important role in the development of various tumors, promoting tumor proliferation and reducing the sensitivity of tumors to 5-fluorouracil in hepatocellular carcinoma^12^. In non-small cell lung cancer, high Notch2 mRNA expression predicted better overall survival in lung adenocarcinoma^13^. Wang^14^ found that overexpression of Notch2 in ESCC is closely related to overall survival (OS) and progression-free survival (PFS) and that its expression could serve as a biomarker to identify individuals with poor prognostic potential. However, current research rarely addresses miRNAs and Notch2 interactions in ESCC.

In this study, we examined the expression of miR-1 in ESCC tissues and plasma and up-regulated or down-regulated miR-1 expression through cell transfection. We observed the effect of miR-1 on ESCC cell proliferation, cell migration and invasion, searched for the direct target gene of miR-1, and explored the mechanism of miR-1 in the pathogenesis of ESCC. The results provide a new theoretical basis for diagnosing and treating ESCC.

## Results

### miR-1 is down-regulated in ESCC tissue and plasma

**Figure 1:**
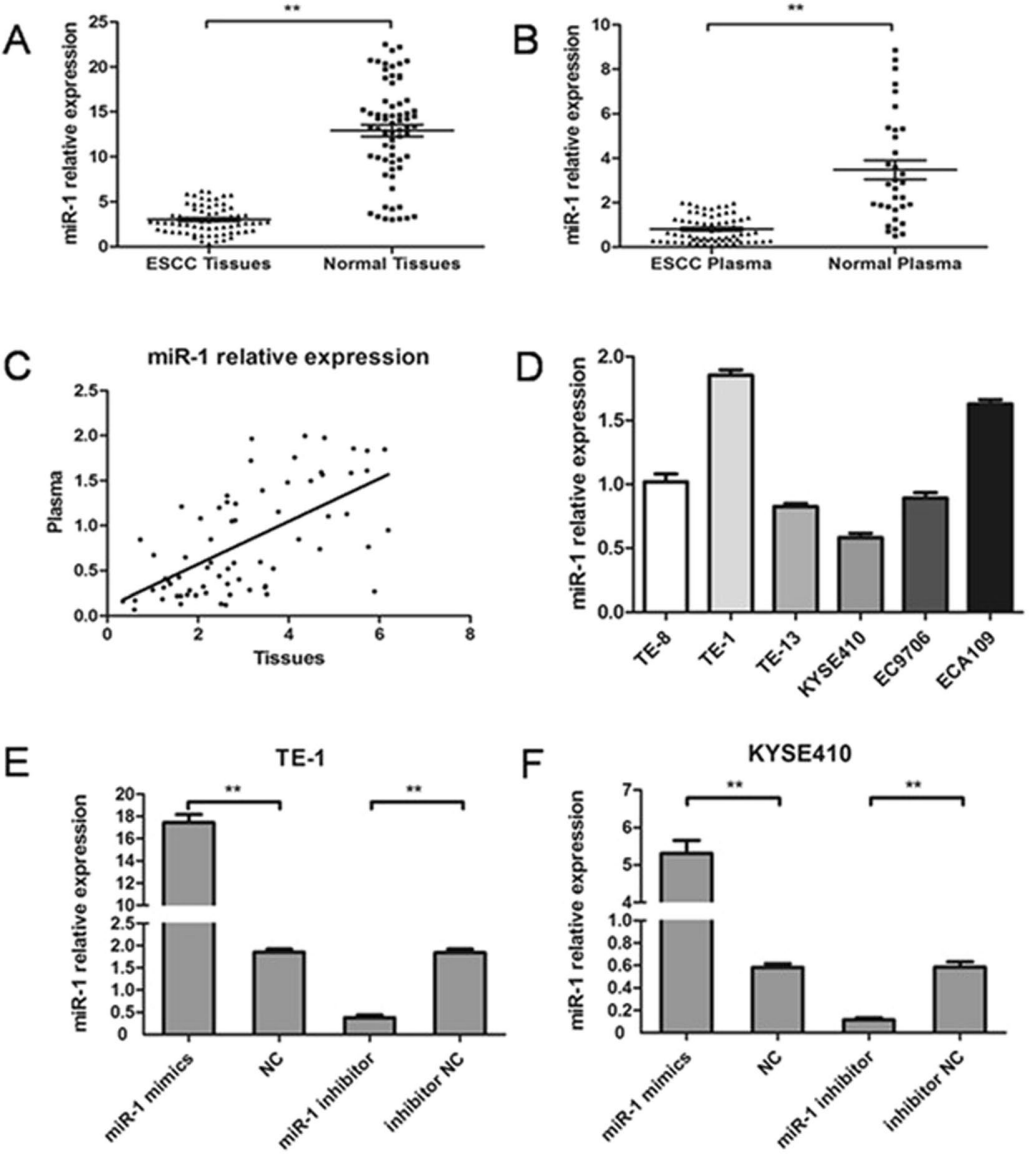
The miR-1 level in tissues, preoperative plasma and ESCC cell lines. (**A**) 69 cases of ESCC tissues were compared with corresponding adjacent normal tissues, and miR-1 in ESCC tissues was significantly lower than that in adjacent normal tissues (**p < 0.01). (**B**) The preoperative plasma of the same 69 patients with ESCC were compared with plasma from 33 healthy volunteers, and the expression level of miR-1 in patients was significantly lower (**p < 0.01). (**C**) miR-1 in the tissues of patients with ESCC was positively correlated with that in preoperative plasma (r = 0.622, p < 0.01). (**D**) The expression level of miR-1 in six ESCC cell lines was determined by qRT-PCR. (**E**,**F**) miR-1 expression levels changed after transfection of TE-1 and KYSE410 cell lines.

Expression of miR-1 in 69 ESCC tissues and corresponding adjacent normal tissues was detected by quantitative real-time PCR (qRT-PCR). The expression level of miR-1 in ESCC tissues was significantly lower than that in adjacent normal tissues (3.004 ± 0.185 vs. 12.886 ± 0.649; p < 0.01) (Fig. 1A). As shown in Table 1, we found that the miR-1 level in tissues was closely related to invasion, lymph node metastasis and TNM staging (p < 0.05). At the same time, we also detected the expression of miR-1 in preoperative plasma from 69 patients and in plasma from 33 healthy volunteers. The miR-1 level in tumor patients was significantly lower than that in healthy volunteers (0.810 ± 0.071 vs. 3.477 ± 0.427; p < 0.01) (Fig. 1B). As shown in Table 1, the expression of miR-1 in plasma was closely related to invasion, lymph node metastasis and TNM staging (p < 0.05). Pearson’s correlation test showed that the level of miR-1 in tumor tissue was positively correlated with that in plasma (r = 0.622, p < 0.01, Fig. 1C).

**Table 1:** The relationship between the levels of miR-1 in tissue and plasma and clinicopathologic characteristics of ESCC patients (*indicates p < 0.05, **indicates p < 0.01).

Characteristics	Number	Tissue	P value	Plasma	P value
Gender					
Male	46	2.962 ± 0.223	0.752	0.799 ± 0.078	0.823
Female	23	3.088 ± 0.336		0.833 ± 0.147	
Age					
≤60	30	3.226 ± 0.290	0.295	0.800 ± 0.108	0.896
>60	39	2.833 ± 0.239		0.818 ± 0.095	
Tumor location					
Upper	14	3.078 ± 0.420	0.979	0.948 ± 0.131	0.625
Middle	28	2.997 ± 0.298		0.777 ± 0.112	
Lower	27	2.973 ± 0.295		0.773 ± 0.123	
Diameter					
≤5 cm	37	2.893 ± 0.263	0.521	0.799 ± 0.092	0.864
>5 cm	32	3.133 ± 0.260		0.823 ± 0.111	
Invasion					
T1	22	3.926 ± 0.331	0.004**	1.107 ± 0.140	0.032*
T2	24	2.681 ± 0.229		0.700 ± 0.097	
T3	20	2.549 ± 0.353		0.661 ± 0.127	
T4	3	1.868 ± 0.832		0.508 ± 0.168	
Lymph node status					
N0	35	3.441 ± 0.251	0.015*	0.963 ± 0.096	0.027*
N1	34	2.555 ± 0.253		0.652 ± 0.098	
TNM stage					
I	14	4.091 ± 0.392	0.001**	1.230 ± 0.163	0.002**
II	39	3.016 ± 0.225		0.782 ± 0.087	
III	16	2.024 ± 0.326		0.511 ± 0.120	
Differentiation					
High	24	3.474 ± 0.322	0.179	0.679 ± 0.101	0.230
Moderate	30	2.761 ± 0.257		0.845 ± 0.147	
Low	15	2.738 ± 0.424		0.829 ± 0.086	
Smoking					
Yes	43	3.108 ± 0.230	0.474	0.829 ± 0.086	0.734
No	26	2.832 ± 0.312		0.779 ± 0.124	
Alcohol drinking					
Yes	48	3.056 ± 0.218	0.676	0.837 ± 0.087	0.570
No	21	2.886 ± 0.355		0.749 ± 0.123	

### miR-1 inhibits cell proliferation, migration and invasion

We detected the miR-1 level by qRT-PCR in six ESCC cell lines (Fig. 1D). TE-1 cells were found to have the highest level of miR-1, and KYSE410 cells expressed the lowest level. Therefore, these two cell lines were used for functional testing. TE-1 and KYSE410 cells were transfected with miR-1 mimics, negative control (NC), miR-1 inhibitor, and inhibitor NC. As shown in Fig. 1E,F, using qRT-PCR, we found that the miR-1 level in the miR-1 mimics group was significantly higher than that in the NC group in both TE-1 and KYSE410 cells. In addition, miR-1 expression in the miR-1 inhibitor group was significantly lower than that in the inhibitor NC group.

**Figure 2:**
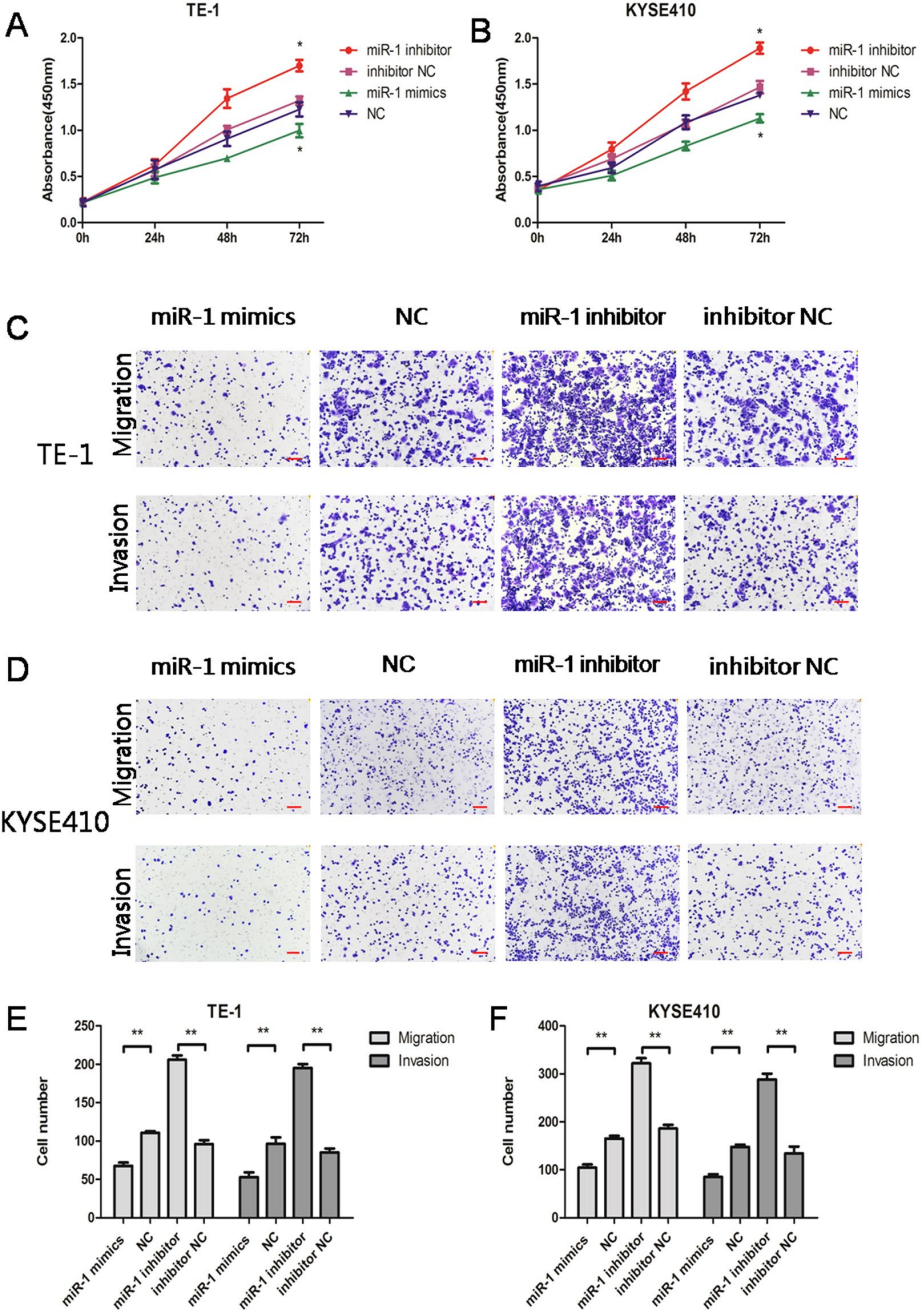
miR-1 inhibits proliferation, migration and invasion in TE-1 and KYSE410 cells. (**A**,**B**) The proliferation ability of cells in the miR-1 mimics group was lower than that of cells in the NC group (*p < 0.05). The proliferation of cells in the miR-1 inhibitor group was significantly higher than that in the NC group (*p < 0.05). (**C**,**E**) The migration and invasion ability of TE-1 cells were different from those of the NC group. Scale bars indicate 100 μm (**p < 0.01). (**D**,**F**) The migration and invasion ability of transfected KYSE410 cells were significantly different from those of the NC group. Scale bars indicate 100 μm (**p < 0.01).

CCK-8 experiments were used to assess the proliferation of ESCC cells at 24, 48, and 72 h after transfection. As shown in Fig. 2A,B, the proliferation of cells in the miR-1 mimics group was significantly lower than that in the NC group for both of TE-1 and KYSE410 cells at 72 h (p < 0.05). In addition, the proliferation ability of cells in the miR-1 inhibitor group was significantly higher than that of cells in the inhibitor NC group (p < 0.05). These results show that miR-1 can inhibit the proliferation of ESCC cells.

As shown in Fig. 2C,D, the migration and invasion of TE-1 and KYSE410 cells in the miR-1 mimics group decreased significantly compared with that in the NC group (p < 0.01), while the migration and invasion of cells in the miR-1 inhibitor group increased significantly compared with the inhibitor NC group (p < 0.01). Therefore, miR-1 can inhibit the migration and invasion of ESCC cells.

### miR-1 directly regulates Notch2 expression by targeting its 3′-UTR

**Figure 3:**
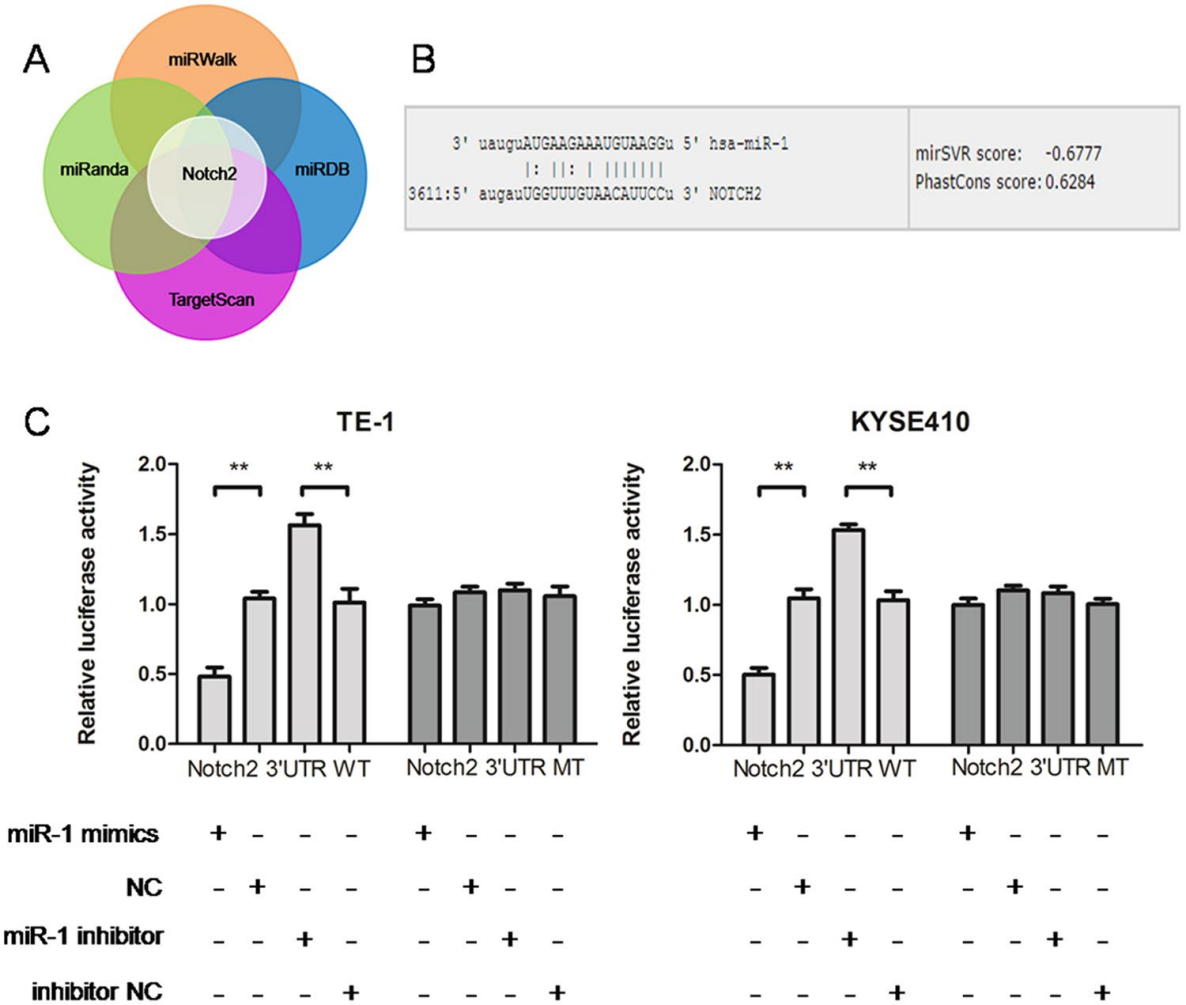
The miR-1 direct target gene was predicted and validated. (**A**) Multiple bioinformatics software predicted that Notch2 is a direct target gene of miR-1. (**B**) miR-1 inhibits Notch2 expression by directly targeting its 3′-UTR. (**C**) A dual-luciferase experiment validated Notch2 as a direct target gene of miR-1.

miRanda, miRDB, miRWalk, and TargetScan were used to predict the potential target gene of miR-1. As shown in Fig. 3A, as a result of the intersection of the four bioinformatics software programs, we predicted that Notch2 may be the target gene for miR-1. In Fig. 3B, we can see that the relationship between Notch2 and miR-1 3′-UTR regions followed the base complementary pairing principle. As shown in Fig. 3C (the dual-luciferase reporter assay), we found that luciferase activity in miR-1 mimic was significantly lower than that of cells transfected NC in the Notch2–3′UTR-WT group (**p < 0.01), but that was no significant difference in the Notch2–3′UTR-MT group, so miR-1 mimic had no effect on Notch2–3′UTR-MT (p > 0.05). Luciferase activity was significantly higher in miR-1 inhibitor than that for cells transfected with inhibitor NC in the Notch2–3′UTR-WT group (**p < 0.01). However, luciferase activity showed no significant difference in the Notch2–3′UTR- MT group (p > 0.05). These observations demonstrate that miR-1 directly regulates Notch2 expression by targeting its 3′-UTR.

**Figure 4:**
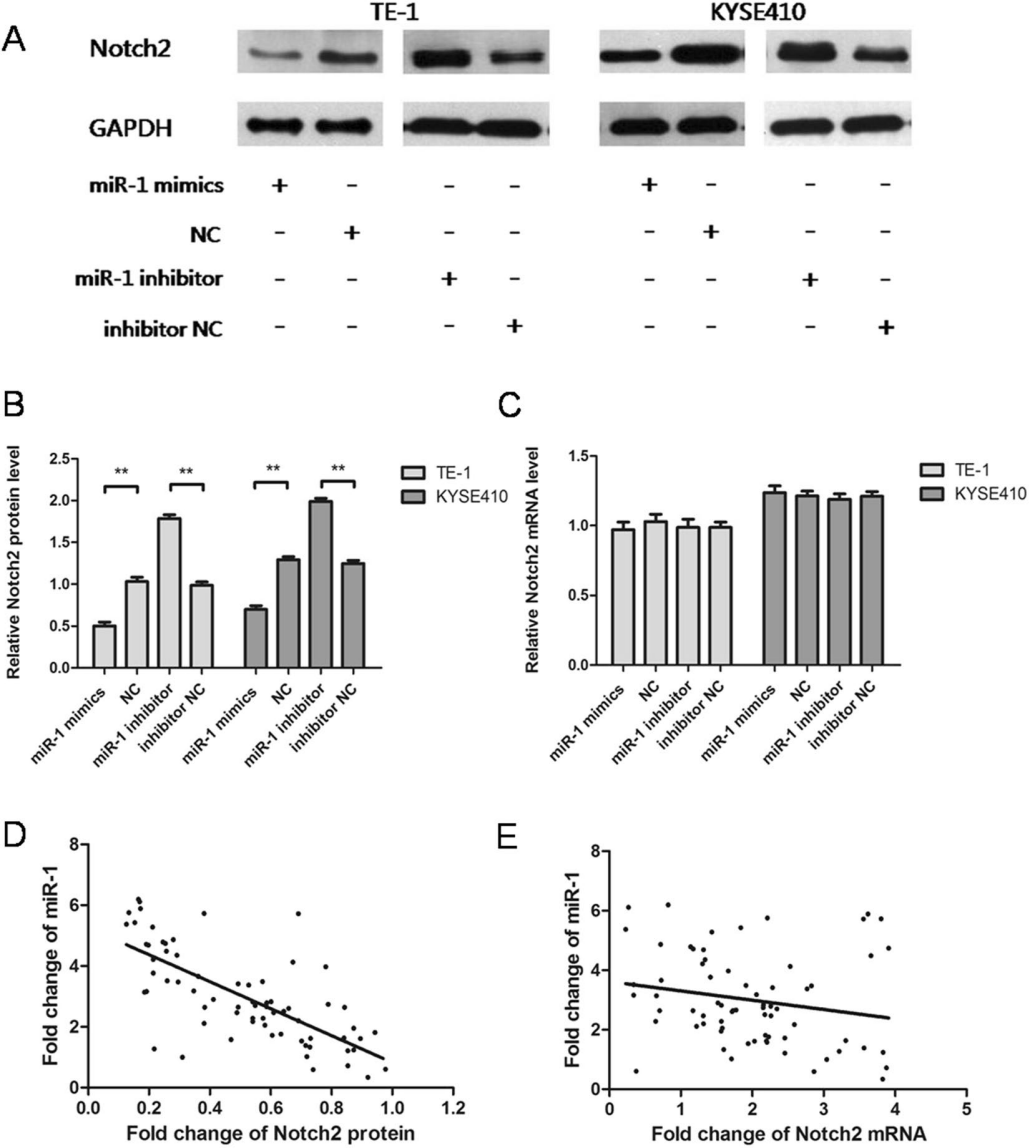
miR-1 post-transcriptionally regulates Notch2 by binding its 3′-UTR. (**A**,**B**) Notch2 protein was altered after transfection of TE-1 and KYSE410 cells. (**C**) Notch2 mRNA did not change significantly after transfection of TE-1 and KYSE410 cells. (**D**) miR-1 and Notch2 protein expression were negatively correlated in ESCC tissues(r = −0.713, p < 0.01). (**E**) miR-1 and Notch2 mRNA expression was not correlated in ESCC tissues (r = −0.202, p = 0.097).

The mechanism of miR-1 regulation of Notch2 was further investigated by assessing expression of miR-1 in transfected TE-1 and KYSE410 cells. Expression of Notch2 protein in the miR-1 mimics group was lower than that in the NC group (Fig. 4A,B), but there was no significant difference in Notch2 mRNA expression (Fig. 4C). Expression of Notch2 protein in the miR-1 inhibitor group was higher than that in the NC group (Fig. 4A,B), but there was no significant difference in Notch2 mRNA expression (Fig. 4C). In ESCC tissue, we found that miR-1 was negatively correlated with Notch2 protein based on a Pearson correlation analysis (r = −0.713, p < 0.01) (Fig. 4D), but not with Notch2 mRNA (r = −0.202, p = 0.097) (Fig. 4E). Thus, these results demonstrate that miR-1 post-transcriptionally regulates Notch2 expression.

### Notch2 expression attenuates the inhibitory effects of miR-1

**Figure 5:**
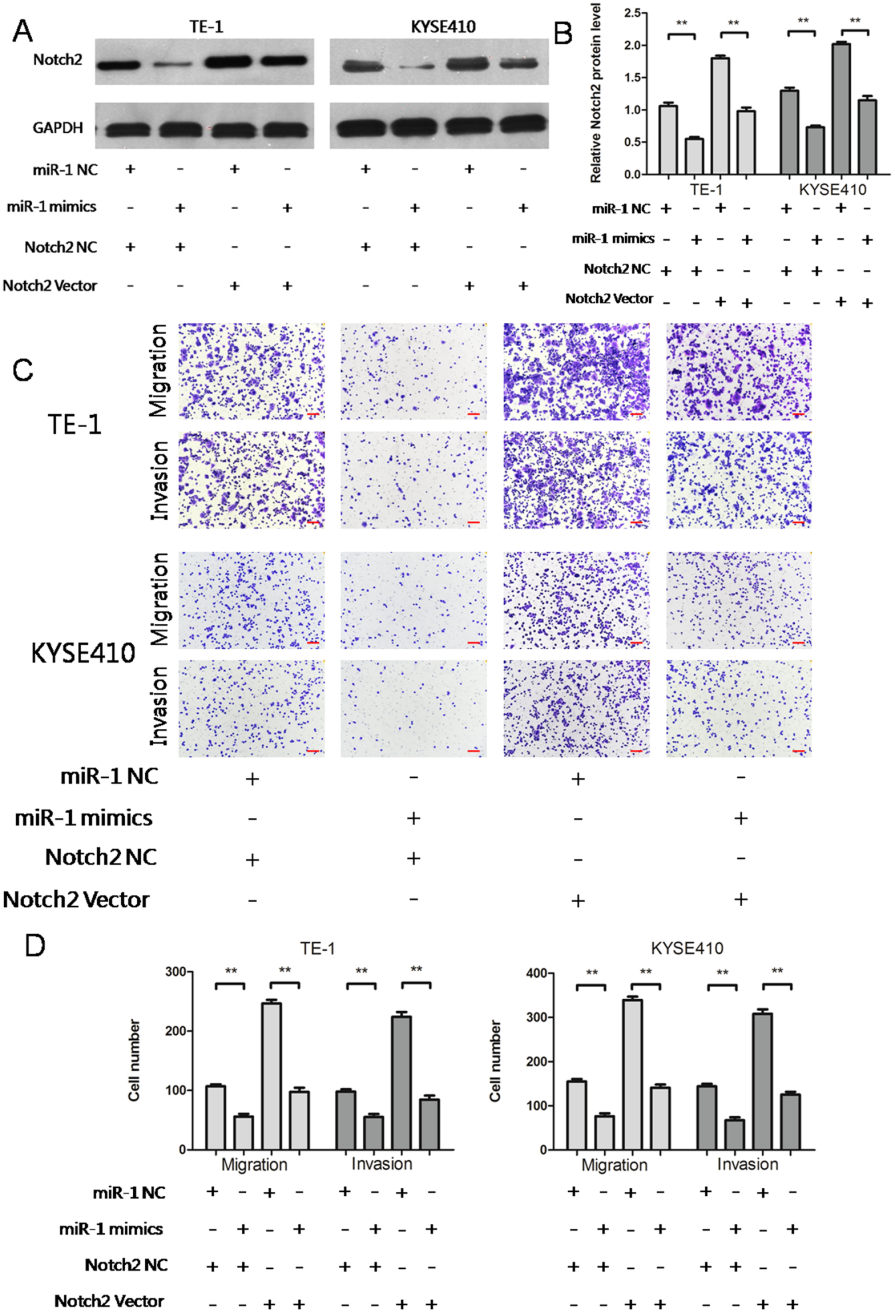
Notch2 expression attenuates the inhibitory effects of miR-1. (**A**,**B**) Notch2 vector attenuated the inhibitory effects of miR-1 mimics on Notch2 protein (**p < 0.01). (**C**,**D**) Notch2 vector attenuated the inhibitory effect of miR-1 mimics on tumour cell migration and invasion (**p < 0.01).

We explored the effects of Notch2 on the migration and invasion of ESCC cells. Notch2 was found to play an oncogenic role and increased the migration and invasion of ESCC cells. To further explore the functional relationship between miR-1 and Notch2, we designed a Notch2 vector and co-transfected TE-1 and KYSE410 cells with this vector and miR-1 mimics. It was found that the Notch2 vector attenuated the miR-1 inhibitory effect of miR-1 mimics on Notch2 protein in tumor cells (Fig. 5A,B). The Notch2 vector attenuated the inhibitory effect of miR-1 mimics on migration and invasion (Fig. 5C,D). Thus, miR-1 regulates ESCC cell migration and invasion by acting on Notch2.

### miR-1 regulates EMT signalling

**Figure 6:**
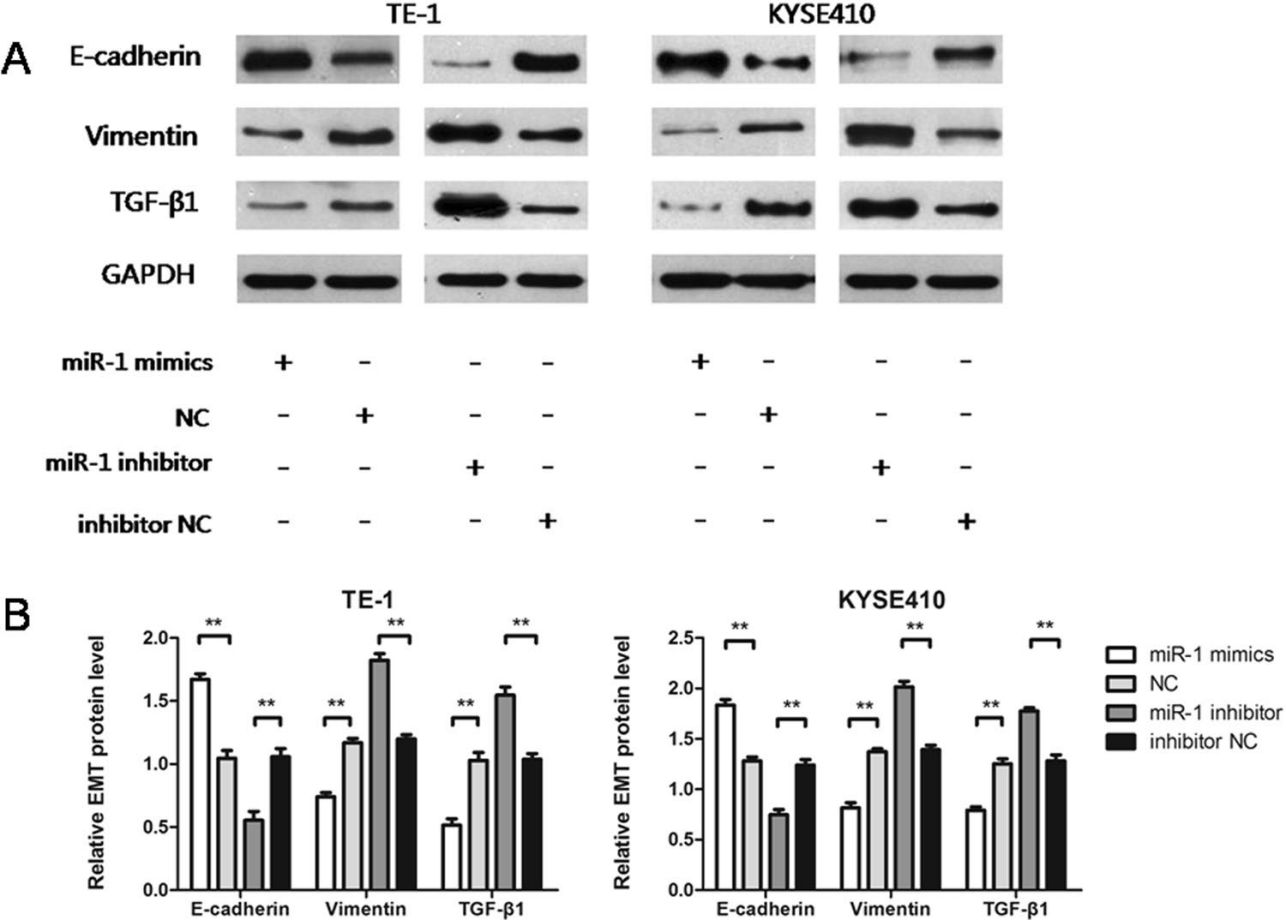
miR-1 regulates EMT signalling. (**A**) E-cadherin, Vimentin, and TGF-β1 protein in the EMT signalling pathway were altered in TE-1 and KYSE410 cells. (**B**) Quantitative changes in E-cadherin, Vimentin and TGF-β1 protein expression after transfection.

To understand whether miR-1 can regulate epithelial-mesenchymal transition (EMT) signalling, we performed transfection experiments to increase or decrease the expression level of miR-1 to observe changes in classical factors, such as E-cadherin, Vimentin and TGF-β1, in the EMT pathway. miR-1 mimics increased E-cadherin protein, while down-regulating the expression of Vimentin and TGF-β1 protein (Fig. 6A,B). miR-1 inhibitor decreased the expression of E-cadherin protein, while Vimentin and TGF-β1 protein was up-regulated (p < 0.01) (Fig. 6A,B). Thus, we speculate that miR-1 can regulate EMT signalling.

## Discussion

miRNAs exert a biological function by targeting the 3′-UTR region of genes. Because this region regulates subcellular localization, nuclear transport and stabilizes transcripts^15^, miRNAs play an important role in cells. A variety of miRNAs are abnormally expressed in ESCC patients, and their expression is closely related to clinical features and tumor biology, such as pathological type, differentiation, lymph node metastasis, TNM staging, prognosis and chemotherapy sensitivity^16–20^. Study of the pathogenesis of miRNAs and ESCC not only provides new clues for molecular markers of ESCC but also provides a useful way to find new drug targets for ESCC.

miR-1 is an important miRNA that acts as a tumor suppressor gene in a variety of malignant tumors. miR-1 is involved in post-transcriptional regulation of crucial tumor-associated genes and is a promising target for anticancer therapy. Re-expression of miR-1 inhibited cancer cell proliferation, promoted apoptosis, and reversed drug resistance in cancer both *in vitro* and *in vivo*^21^. Overexpression of miR-1 in prostate cells led to growth inhibition and down-regulation of genes in pathways that regulate cell cycle progression, mitosis, and DNA replication/repair and participate in dynamics. miR-1 acted as a tumor suppressor in prostate cancer by affecting a variety of cancer-related processes and by inhibiting cell proliferation and movement^22,23^. miR-1 inhibited proliferation, invasion and migration of bladder cancer cells by up-regulating SFRP1 expression^24^. miR-1 could suppress tumor growth and metastasis in gastric cancer and breast cancer and could also prolong the G1 phase^25^. miR-1 also played the role of a tumor suppressor gene in ESCC and can inhibit tumor proliferation, invasion and metastasis^11,26^. However, the mechanism of carcinostasis is still unclear, and it merits further exploration.

We confirmed low expression of miR-1 in ESCC tumor tissue and first detected low expression of miR-1 in preoperative ESCC plasma, and the miR-1 levels in plasma and tissues were associated with invasion, lymph node metastasis and TNM staging. In addition, miR-1 expression in tissues was consistent with that in plasma. Therefore, miR-1 may be a potential tumor marker for diagnosis of ESCC. miR-1 is also an important factor in the diagnosis and prognosis of breast cancer and liver cancer^27,28^. Circulating miR-1 has the potential to be a new biomarker of doxorubicin-induced cardiotoxicity in breast cancer patients^29^.

We further investigated the function of miR-1 in two representative cell lines: TE-1 and KYSE410 cells. We transfected mimics or inhibitor into cells, thus increasing or decreasing the miR-1 level. An increase in miR-1 was found to inhibit the proliferation, migration and invasion of TE-1 and KYSE410 cells, while decreased miR-1 expression enhanced the proliferation, migration and invasion of tumor cells. In osteosarcoma, miR-1 exerts an inhibitory effect on cell proliferation and invasion by acting on VEGFA^30^. miR-1 overexpression promoted apoptosis by targeting G6PD in cervical cancer cells^31^.

The relationship between miRNA and target genes is complex. The same miRNA typically regulates multiple target genes^32,33^, and multiple miRNAs can act on the same target gene^34,35^. In recent years, the active involvement of miR-1 in regulation of various target genes has been investigated. miR-1 expression levels were found to be significantly correlated with tumor invasion and advanced clinical stage^36^. miR-1 suppresses cell proliferation, invasiveness, metastasis, and ESCC progression by binding to its target genes LASP1 and TAGLN2^26^. To further elucidate the carcinogenesis mechanism of miR-1, we first took advantage of bioinformatics software, combined with tumor invasion and metastasis related genes in ESCC, and selected Notch2 as the potential target gene. As a major receptor in the Notch pathway, Notch2 plays an important role in the development and progression of acute myeloid leukaemia, bladder cancer, gastric cancers, ESCC and other malignant tumors^14,37–39^. We found that miR-1 can down-regulate Notch2 protein in tumor cells, but no change in Notch2 mRNA was observed. The miR-1 in tumor tissue was correlated with Notch2 protein level, but not with Notch2 mRNA. We found that Notch2–3′UTR-WT could significantly decrease the luciferase activity of miR-1-overexpressing ESCC cells and increase the luciferase activity of ESCC cells with low expression of miR-1. We found that Notch2 vector was able to attenuate the inhibitory effect of miR-1 mimics on Notch2 protein, migration and invasion in ESCC cells. It was further verified that miR-1 inhibited growth and invasion of tumors by regulating Notch2 at the post-transcriptional level.

EMT refers to morphological changes that occur in cells during transition from an epithelial to an interstitial cell phenotype. Epithelial cells lose cell polarity and intercellular adhesion and access migration and invasion ability, developing mesenchymal cell morphology and characteristics. EMT plays an important role in tumor invasion and metastasis. A variety of signalling pathways are involved in EMT in tumor cells, such as the Notch, Wnt, TGF-β, NF-κB, and other pathways. This study found that miR-1 increased E-cadherin protein and down-regulated the expression of Vimentin and TGF-β1 protein. Therefore, we hypothesized that miR-1 regulates EMT signalling directly through Notch2.

The expression of miR-1 in ESCC tissues and peripheral plasma was significantly decreased, and its expression level was closely related to invasion, lymph node metastasis and TNM stage. As a tumor suppressor gene, miR-1 inhibited ESCC cell proliferation, migration and invasion. It can inhibit the development and progression of ESCC by directly regulating the expression of Notch2 protein. miR-1 can be used as a potential tumor marker for early diagnosis of ESCC and a new drug target.

## Materials and Methods

### Clinical Specimens

A total of 69 patients were first diagnosed with ESCC at Taian Central Hospital (Taian, Shandong, China) from June 2014 to May 2016. Preoperative patients did not receive radiotherapy, chemotherapy, biological therapy, or traditional Chinese medicine treatments. Peripheral blood (2 ml) was collected from each patient before surgery. ESCC tissues, adjacent normal tissues from the same patients and plasma samples from 33 healthy volunteers were also collected. This study was approved by the Ethics Committee of Taian Central Hospital, and each patient signed an informed consent. The study was based on the Declaration of Helsinki.

### Cell Culture

The ECA109, TE-1, TE-8, and TE-13 cell lines were donated by the Central Laboratory of Shandong Cancer Hospital Affiliated to Shandong University (Jinan, Shandong, China). The KYSE410 and EC9706 cell lines were purchased from the Shanghai Cell Bank of Chinese Academy of Medical Sciences (Shanghai, China). Six ESCC cell lines were cultured in RPMI-1640 medium (HyClone, Logan, Utah, USA) containing 10% foetal bovine serum (FBS, HyClone) and 0.2% penicillin streptomycin (Invitrogen, Carlsbad, CA, USA). All cells were cultured in a 5% CO_2_ incubation chamber at 37 °C.

### RNA extraction, reverse transcription and qRT-PCR

The miRNeasy Mini Kit (Qiagen, Duesseldorf, Nordrhein-Westfalen, Germany) was used to extract the total RNA from ESCC tissues, adjacent normal tissues and ESCC cells. The miRNeasy Serum/Plasma Kit (Qiagen) was used to extract the total RNA from serum collected from ESCC patients before surgery and from healthy volunteers. RNA integrity was detected by formaldehyde denaturing agarose gel electrophoresis. The purity of the total RNA was detected by the A260/280 ratio. The miScript II RT Kit (Qiagen) was applied for reverse transcription. A miScript SYBR Green PCR Kit (Qiagen) was used to perform PCR, and U6 was used as the internal reference to normalize the target gene levels. The relative values for miR-1 were calculated by the 2^−ΔΔCt^ method. SYBR Green PCR Mix (Aidlab, Beijing, China) was used to analyze Notch2 mRNA expression using qRT-PCR. GAPDH levels were assessed to standardize the expression of Notch2 mRNA levels. The relative value of Notch2 was calculated with the 2^−ΔΔCt^ method. The following primers were used for reverse transcription and qRT-PCR: (1) miR-1-forward 5′-CAGTGCGTGTCGTGGAGT-3′, (2) miR-1-reverse 5′-GGCCTGGAATGTAAAGAAGT-3′, (3) Notch2-forward 5′-GGGACCCTG- TCATACCCTCT-3′, (4) Notch2-reverse 5′-GAGCCATGCTTACGCTTTCG-3′.

### Cell transfection

miR-1mimics, miR-1 inhibitor, NC and inhibitor NC were designed and synthesized by GenePharma (Shanghai, China). Solutions were dissolved in DEPC water to reach a final concentration of 20 µM. In a six-well plate, approximately 5–6 × 10^5^ cells in logarithmic growth phase in medium containing serum and double antibody were added to each well. miR-1 mimics, NC and serum-free 1640 medium were added to TE-1 and KYSE410 cells to a final concentration of 50 nM. miR-1 inhibitor, inhibitor NC and serum-free medium were added to TE-1 and KYSE410 cells to a final concentration of 150 nM. For each group, 12 μl of HiPerFect transfection reagent was added to the samples. After incubation at room temperature for 5–10 min, the mixture was added to the cells, and the cells were placed in an incubator.

### Cell proliferation assay

The cell suspension was placed in a 96-well plate with approximately 3–4 × 10^3^ cells per well. After transfection for 24, 48, or 72 h, 10 μl of Cell Counting Kit-8 (CCK-8, Beyotime, China) reagent was added to each well. The 96-well plate was placed in a 37 °C, 5% CO_2_ incubator, and the absorbance was measured at 450 nm using a microplate reader after 2 h.

### Cell migration and invasion assay

After transfection for 24 h, cells were cultured in serum-free medium for 12 h. The cell concentration was adjusted to 4–5 × l0^5^/ml. A transwell chamber with 8-µm pores (Corning, Corning, NY, USA) was used for the 24-well plates. RPMI-1640 medium (500 μl) containing 10% FBS was placed in the lower layer, and 200 μl of the cell suspension was placed in the upper chamber. Then, the cells were incubated for 10 h. The cells on the lower surface of the chamber were fixed with glacial acetic acid for 15–30 min and stained with crystal violet for 30 min, and 10 fields were selected randomly to count. The cells were covered with Matrigel (BD, Franklin, NJ, USA), and the same method was used to perform cell invasion assays.

### Western blot

Lysate was used to extract the total protein from the tissues and cells. The protein concentration of each sample was measured and adjusted to a uniform concentration. The protein samples were separated on 10% SDS polyacrylamide gels (SDS-PAGE) and transferred to polyvinylidene fluoride membranes at 100 V for 2.5 h. The membrane was blocked with 5% fat-free milk in TBST and incubated with primary antibodies (Abcam, Cambridge, UK) (anti-Notch2, 1: 500; anti-E-cadherin, 1: 500; anti-Vimentin, 1: 1000; and anti-TGF-β1, 1: 1000) at 4 °C overnight. Then, secondary antibodies (1:5000) were added for 2 h at room temperature. The results were visualized using chemiluminescence (Millipore, MA, USA). Image J software (National Institutes of Health, Bethesda, USA) was used to detect the protein expression levels. GAPDH (1:1000) was used as the control.

### Luciferase reporter assay

The 3′-UTR sequence of wild-type Notch2 and that of a target-site mutant were amplified by PCR, cloned into a dual-luciferase reporter plasmid (Promega, Madison, WI, USA), and named pGL3-Notch2–3′-UTR-WT (wild-type vector) and pGL3-Notch2–3′-UTR- MUT (mutant vector). TE-1 and KYSE410 cells in logarithmic growth phase were inoculated into 96-well plates at 1.5 × 10^4^ cells per well before transfection. TE-1 and KYSE410 cells were co-transfected with the Wt or Mut vector and miR-1 mimics, NC, miR-1 inhibitor, or inhibitor NC using the Attractene Transfection Reagent (Qiagen). The ratio of firefly to Renilla luciferase activity was measured with a dual-luciferase reporter system (Promega, Madison, WI, USA) after transfection for 48 h.

### Statistical analysis

Statistical analysis was performed using the SPSS 17.0 software. The results are presented as the mean ± SEM. A T-test was used to detect differences between the different groups (ESCC tissue, adjacent normal tissue, ESCC plasma, and healthy volunteer plasma). ANOVA was used to assess miR-1 expression under different clinicopathological features. Pearson analysis was used to assess the relationship between miR-1 expression and the clinicopathological features. The T-test was used to analyze cell experiments. p < 0.05 was considered statistically significant.
